# Incidental Finding of a Right Atrial Appendage Thrombus in a Patient With Dilated Cardiomyopathy and Diffuse B-cell Lymphoma

**DOI:** 10.7759/cureus.71381

**Published:** 2024-10-13

**Authors:** Aakash Rana, Jack Xu, Rupesh Manam

**Affiliations:** 1 Medicine, Central Arkansas Veterans Healthcare System, Little Rock, USA; 2 Cardiology, Novant Health, Winston-Salem, USA

**Keywords:** atrial flutter, diffuse b-cell lymphoma, dilated cardiomyopathy, intracardiac thrombi, right atrial appendage thrombus, right atrial thrombus

## Abstract

Right atrial thrombus is a rare phenomenon linked with a high risk of mortality. We present a case of a 75-year-old male with dilated cardiomyopathy and B-cell lymphoma on chemotherapy via an implanted chemo port who presented with dyspnea secondary to new-onset atrial flutter. During the evaluation for new-onset atrial flutter, a thrombus in the right atrial appendage was incidentally found on transesophageal echocardiogram (TEE). This finding was confirmed with cardiac magnetic resonance imaging. A multidisciplinary heart team evaluation was performed, and the patient was deemed not a surgical candidate. The patient was discharged on apixaban for the right atrial thrombus treatment and later deferred to hospice care. This case highlights that there are no formal guidelines for managing right atrial thrombi (RAT), but treatment options include anticoagulation, thrombolytic therapy, and surgical thrombectomy or embolectomy with vacuum extraction. Additional research is needed to develop appropriate guidelines for management and prevent further systematic complications.

## Introduction

Right atrial thrombus is rare in clinical practice, associated with an overall mortality rate of 28%. Among untreated patients, the mortality rate can reach 80-100% [1,2]. Right atrial thrombi (RAT) are commonly seen in patients with atrial fibrillation/flutter, foreign bodies inside the atrium such as central venous catheters and pacemakers leads, emboli of deep venous thrombus, and primary or metastatic tumors [3]. RAT are divided into three groups: Type A (RAT in transit), Type B (RAT in situ), and Type C (mobile in situ thrombi) [4]. Type A RAT is serpiginous, highly mobile, originating from the deep venous system, less intracardiac abnormalities, and requires emergency treatment. Type B RAT is less mobile, pedunculated, broad-based with an underlying intracardiac abnormality, more chances of embolization but a better prognosis. Type C RAT has a stalk and a thin point of attachment to the atrial wall, similar to atrial myxoma [4]. Treatment options for RAT include anticoagulation, thrombolytic therapy, and surgical thrombectomy/embolectomy with vacuum extraction [5,6]. We present a case report of incidental discovery of right atrial appendage thrombus in a patient with dilated cardiomyopathy, B-cell lymphoma, implanted chemo port, and newly diagnosed atrial flutter.

## Case presentation

A 75-year-old male with a past medical history of coronary artery disease, dilated cardiomyopathy, chronic myeloid leukemia in remission, prostate cancer in remission, stage 4 diffuse B-cell lymphoma on current chemotherapy via implanted chemo port, hypertension, chronic kidney disease stage 3a and hyperlipidemia who presented to the emergency department (ED) for dyspnea. The patient had an episode of dyspnea while traveling over the last several months, and approximately 1 to 2 weeks ago began having progressive dyspnea on exertion. This progressed to the point that he was unable to walk across the room without significant dyspnea. Vital signs: blood pressure 113/81 mm HG, heart rate 150 beats per min, and saturation of 97% on room air. On physical exam, the patient was tachycardic in regular rhythm with 2+ pitting edema of the lower extremities. Labs obtained in the ED (Table 1).

**Table 1:** Admission laboratory results of the patient

Blood Analysis	Results	Reference range	Units
Hemoglobin	10.7	12-15	(g/dl)
Platelets	237	150-150	(×10^3^ cells/µL)
Sodium	134	130-145	mmol/L
Admission creatinine	2.90	0.5-1.2	mg/dl
Baseline creatinine	2.00	0.5-1.2	mg/dl
Blood urea nitrogen	68	2-20	mg/dl
Aspartate aminotransferase	62	5-30	IU/L
Alanine aminotransaminase	58	5-55	IU/L
High-sensitivity cardiac troponin on arrival	150	<14	ng/L
High-sensitivity cardiac troponin at 3 hour	137	<14	ng/l

**Figure 1:**
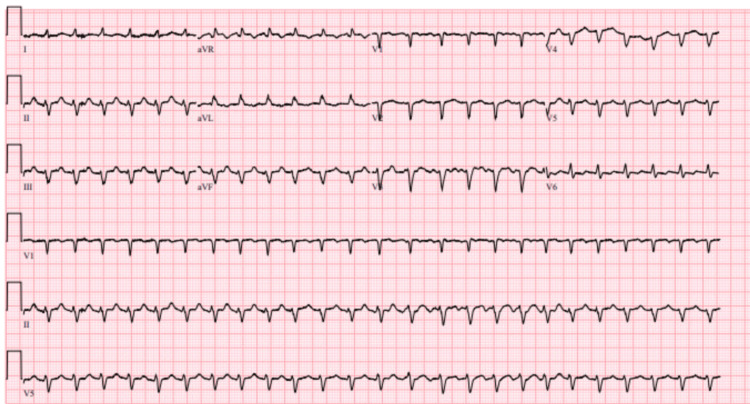
Electrocardiogram (ECG) showed a narrow complex tachycardia concerning an atrial flutter with a 2:1 AV block

An electrocardiogram (ECG) showed a narrow complex tachycardia concerning atrial flutter with a 2:1 AV block (Figure 1).

**Figure 2:**
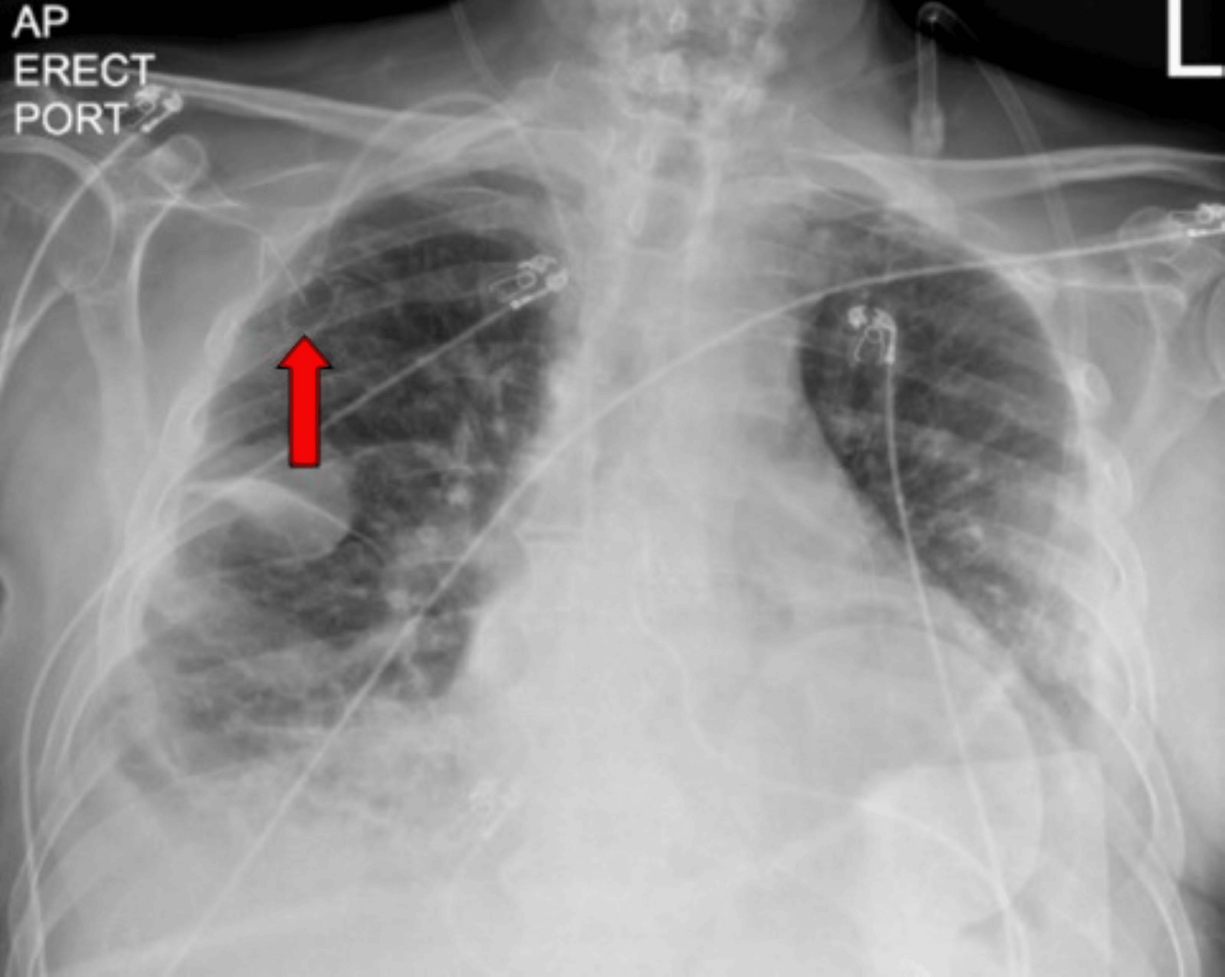
Chest X-ray showed pulmonary edema, a right internal jugular vein chest port (red arrow), and a right lower lobe infiltrate

Chest X-ray showed pulmonary edema, a right internal jugular vein chest port, and a right lower lobe infiltrate (Figure 2).

Transthoracic echocardiography (TTE) showed a left ventricular ejection function of 30-35% with no evidence of thrombus detected in the imaging. Transesophageal echocardiogram (TEE) showed a left ventricular ejection function of 20-25% with several global hypokinesis of the left ventricle, moderate to severe reduced systolic function of the right ventricle, presence of a catheter in the right atrium, and a moderately sized thrombus (1.8 cm x 1.6 cm) in the right atrial appendage (Video 1 and Video 2).

**Video 1:** Transesophageal echocardiogram with a mid-esophageal bicaval view at 130° shows a moderately sized thrombus (1.8 cm x 1.6 cm) in the right atrial appendage

**Video 2:** 3D transesophageal echocardiogram of the right atrial appendage showing the thrombus

**Figure 3:**
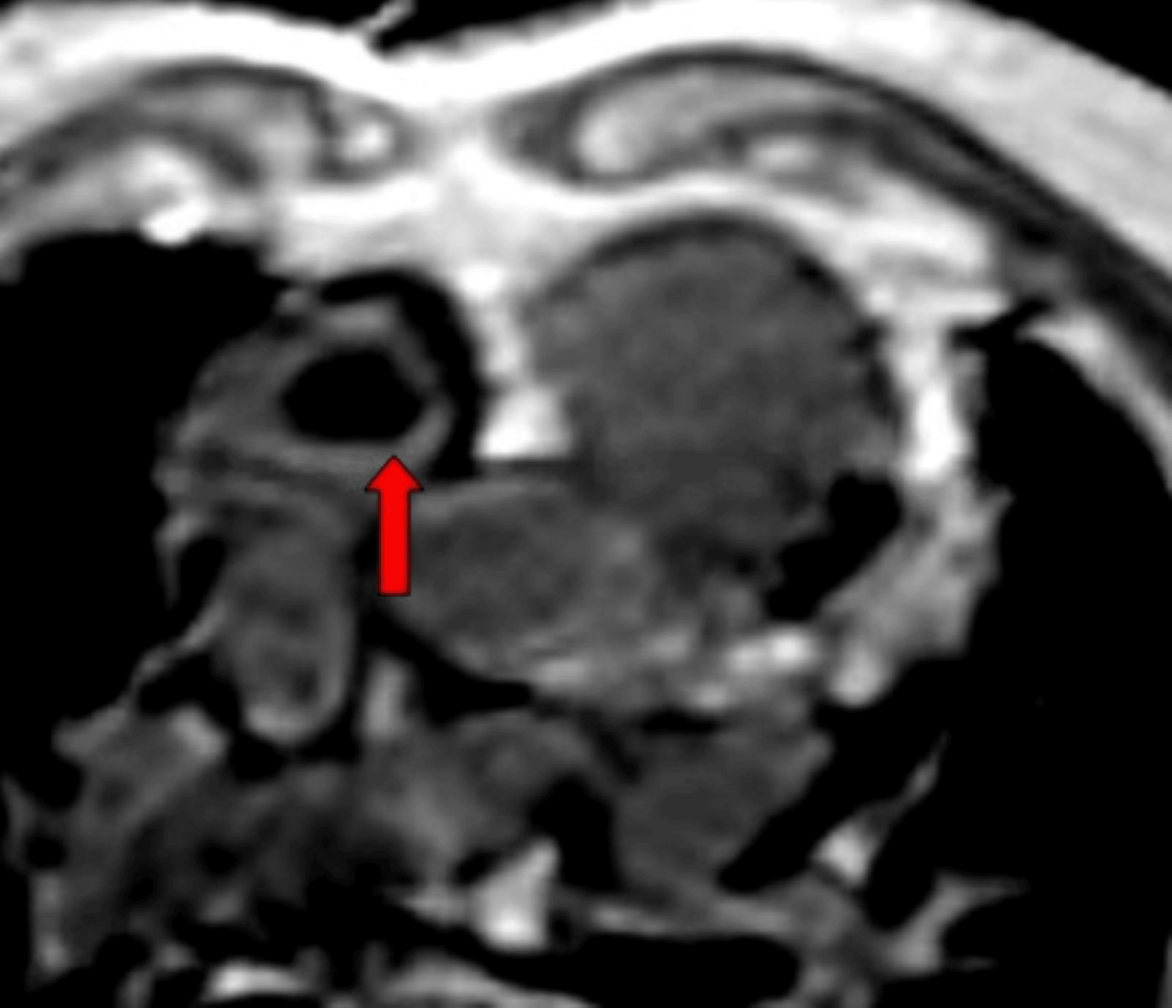
Cardiac magnetic resonance imaging in the four-chamber view shows a 2.1 cm x 1.5 cm mass with no enhancement on a Ti600 sequence consistent with a thrombus

**Figure 4:**
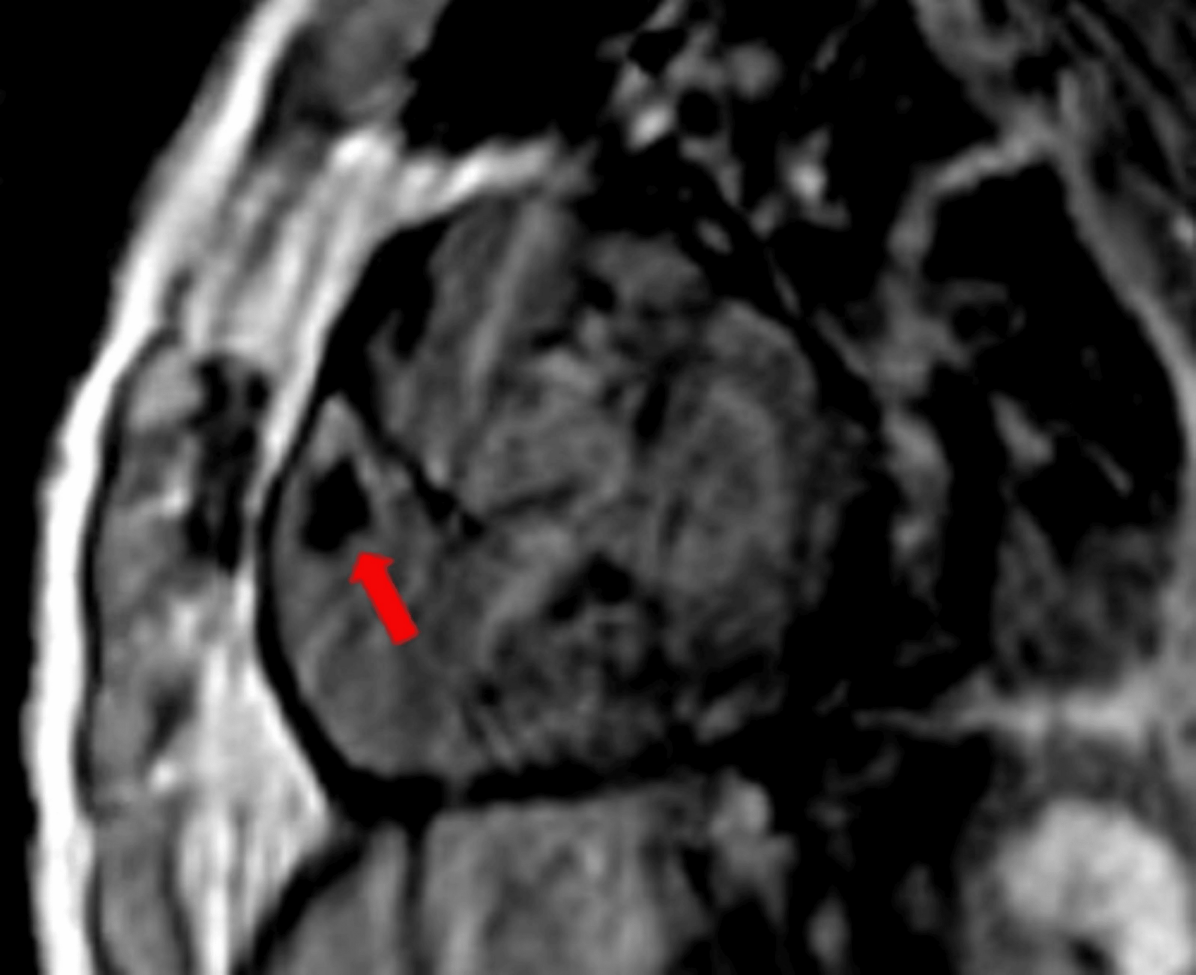
Cardiac magnetic resonance imaging in the short-axis view shows a 2.1 cm x 1.5 cm mass with no enhancement on a Ti600 sequence consistent with a thrombus

These findings were confirmed with cardiac magnetic resonance imaging (MRI), which showed a 2.1 cm x 1.5 cm mass with no enhancement on the Ti600 sequence consistent with a thrombus (Figure 3 and Figure 4).

A multidisciplinary heart team evaluation was performed to determine the best treatment for the right atrial thrombus. Due to medical co-morbidities and current malignancy status, the patient was not a surgical candidate. The patient was discharged from the hospital on apixaban for right atrial appendage thrombus and metoprolol succinate with digoxin for rate control. The patient was supposed to follow up in the cardiology clinic in six weeks with repeat TTE for the right atrial thrombus but decided to go to hospice due to a deteriorating medical status from malignancy.

## Discussion

The differential diagnoses of RA masses are benign or malignant neoplasm, tricuspid valve vegetation, and thrombus [7]. In our case, a right atrium thrombus (RAT) was discovered incidentally during a TEE obtained for atrial flutter workup and confirmed with cardiac MRI. The patient had several predisposing risk factors for RAT such as dilated cardiomyopathy (DCM), diffuse B cell lymphoma, and indwelling chemo-port. We believe the thrombus formation in the right atrium was secondary to 1) dilated cardiomyopathy, 2) diffuse B cell lymphoma, and 3) indwelling chemo-port, all contributing together. Dilated cardiomyopathy (DCM) is characterized by ventricular dilation and dysfunction. Crucially, this dilation originates from left ventricular (LV) remodeling and fibrosis, which leads to myocardial damage in the LV and decreased systolic function [8,9]. As a result, various prothrombogenic factors are affected, including blood stasis and local myocardial injury, which can lead to thrombosis and systematic embolism [10]. We believe that additional risk factors, such as malignancy or the presence of a catheter/pacemaker leads, are necessary in the context of DCM to trigger the formation of RATs since DCM alone is more common to cause thrombus formation in the LV rather than in the right heart. The incident rate of venous thromboembolism in patients with diffuse large B-cell lymphoma is around 10%-12%. Risk factors of cancer-associated thrombosis include surgery, chemotherapy, and central venous catheterization [11]. Patients with malignancy can induce a prothrombotic state since cancer cells can activate the coagulation cascade and induce venous thromboembolism (VTE), such as right atrial thrombus, in our case [11]. Lastly, the presence of the indwelling chemotherapy port may have played a role in the development of the RAT by potentially damaging the atrial wall, even though no thrombus was observed on the catheter's tip. Based on the imaging findings, we believe the right atrial thrombus in our patient should be characterized as a Type B since it is less mobile without a specific shape. The etiology of a Type B RAT is believed to occur due to cardiac risk factors compared to the thrombophilic state caused by malignancy.

Treatment options for RAT include anticoagulation, thrombolytic therapy, and surgical thrombectomy/embolectomy with vacuum extraction. The best therapeutic approach for RAT remains a topic of debate. If the RAT is not treated, it can break loose and travel to the lungs, resulting in a pulmonary embolism. This condition can lead to severe complications such as chronic thromboembolic pulmonary hypertension (CTEPH), right heart failure, and potentially cardiogenic shock [12]. If left untreated, pulmonary embolism can carry a mortality rate of up to 30% [12]. In the European Cooperative Study, the mortality rate was reported to be 60% for anticoagulated patients, 40% for those treated with thrombolytics, and 27% for those submitted to surgical procedures, such as traditional open thrombectomy and percutaneous thrombectomy, which suggests the surgical approach to the most effective [13]. However, the surgical approach is the most invasive and is limited by the scarcity of surgeons with expertise in these procedures [13]. A novel approach to treating RAT consists of using the AngioVac thrombectomy system (AngioDynamics, Inc, Latham, New York, US), a veno-venous filtration apparatus used for the aspiration of thrombi and/or vegetations, which could be used with clinical benefits in patients with right atrial thrombi. However, this system is used at only a few centers, and there is limited research on its effectiveness [14]. In our case, the patient was not a surgical candidate due to medical co-morbidities and current malignancy status and was treated with anti-coagulation with apixaban briefly before the patient elected for hospice care.

## Conclusions

Right atrial thrombi (RAT) are often found incidentally on imaging as presented in our patient. The diagnostic accuracy of different cardiac imaging techniques depends on the location and type of thrombi. Early detection and treatment are necessary to prevent further systematic complications. In our case, the patient decided to go to hospice care six weeks after the diagnosis of RAT with no systematic complications noticed while briefly taking apixaban. There are no formal guidelines for managing RATs, but treatment options include anticoagulation, thrombolytic therapy, and surgical thrombectomy or embolectomy with vacuum extraction, all of which have proven effective in patients with RAT. More studies need to be performed to establish proper guidelines on these subject matter as cardiac imaging improves.
